# Timing of Maternal COVID-19 Vaccine and Antibody Concentrations in Infants Born Preterm

**DOI:** 10.1001/jamanetworkopen.2023.52387

**Published:** 2024-01-19

**Authors:** Alisa Kachikis, Mindy Pike, Linda O. Eckert, Emma Roberts, Yael Frank, Amber L. Young, Erin Goecker, Michael G. Gravett, Alexander L. Greninger, Janet A. Englund

**Affiliations:** 1 Department of Obstetrics and Gynecology, University of Washington, Seattle; 2 Department of Global Health, University of Washington, Seattle; 3 Department of Obstetrics and Gynecology, University of California, San Diego; 4 School of Medicine, Tel Aviv University, Tel Aviv, Israel; 5 Department of Laboratory Medicine, University of Washington, Seattle; 6 Seattle Children’s Hospital Research Institute, Department of Pediatrics, University of Washington, Seattle

## Abstract

**Importance:**

COVID-19 vaccine–derived antibodies in pregnant people may protect infants from severe infection in the first 6 months of life via transplacental antibody transfer. Few data exist on maternally derived SARS-CoV-2 antibodies in preterm compared with full-term infants in association with vaccination timing.

**Objective:**

To compare SARS-CoV-2 anti-Spike (anti-S) antibody levels in preterm and full-term infants in the context of vaccine dose timing before delivery.

**Design, Setting, and Participants:**

This prospective cohort study enrolled pregnant individuals and collected paired maternal and cord blood samples at delivery at the University of Washington between February 1, 2021, and January 31, 2023. Participants who had received at least 2 doses of a messenger RNA COVID-19 vaccine before delivery and did not have a history of prior COVID-19 infection or detectable anti–SARS-CoV-2 nucleocapsid antibodies were included.

**Exposures:**

Timing of the last vaccine dose and preterm or full-term gestational age at delivery.

**Main Outcomes and Measures:**

Paired maternal and cord samples were tested for anti-S antibody, and linear regression was used to evaluate associations between preterm delivery and anti-S antibody levels. Covariates included timing of last dose, number of doses, insurance status, and immunosuppressing medications.

**Results:**

A total of 220 participants (median [IQR] age, 34 [32-37] years; 212 [96.4%] female) with 36 preterm and 184 full-term deliveries were studied. Before delivery, 121 persons received 2 vaccine doses and 99 persons received 3 or more vaccine doses. The geometric mean concentration of maternal anti-S antibodies was 674 (95% CI, 577-787) after 2 doses and 8159 (95% CI, 6636-10 032) after 3 or more doses (*P* < .001). The cord anti-S antibody geometric mean concentration was 1000 (95% CI, 874-1144) after 2 doses and 9992 (95% CI, 8381-11 914) after 3 or more doses (*P* < .001). After adjustment for vaccine timing and number of doses before delivery, no association was found between preterm delivery and cord anti-S antibody levels (β = 0.44; 95% CI, −0.06 to 0.94).

**Conclusions and Relevance:**

In this prospective cohort study of pregnant individuals with preterm and full-term deliveries, receipt of 3 or more compared with 2 doses of COVID-19 vaccine before delivery resulted in 10-fold higher cord anti-S antibody levels. Maternal antibody concentration appeared more important than delivery gestational age in determining cord anti-S antibody levels. The number of doses and timing considerations for COVID-19 vaccine in pregnancy should include individuals at risk for preterm delivery.

## Key Points

**Question:**

Does timing of COVID-19 vaccine administration affect maternally derived anti-Spike antibody concentrations in preterm compared with term infants?

**Findings:**

Among 220 pregnancies in this cohort study, 36 pregnant participants (16.4%) delivered preterm infants before 37 weeks’ gestational age and 184 (83.6%) delivered at full term. After adjustment for timing of last vaccination dose, there was no difference in maternally derived anti-Spike IgG in preterm compared with full-term infants.

**Meaning:**

These findings suggest that maternal anti-Spike antibody concentration is a key factor in determining preterm and full-term infant maternally derived anti-Spike concentrations.

## Teaser

This cohort study compares SARS-CoV-2 antibody levels in preterm compared with full-term infants in the context of vaccine dose timing before delivery.

## Introduction

COVID-19 vaccines have been instrumental in decreasing morbidity and mortality from SARS-CoV-2 infection during pregnancy.^1,2,3^ COVID-19 infection in pregnancy is associated with increased risk of hospitalization, intensive care unit admission, and mortality compared with nonpregnant individuals.^4,5^ Studies^1,6^ completed after the introduction of COVID-19 vaccines have shown that pregnant individuals who received vaccination have lower rates of maternal morbidity, hospital admission, critical care admission, stillbirth, and neonatal demise compared with pregnant individuals who were not vaccinated. COVID-19 vaccines induce anti-Spike (anti-S) antibody production in pregnant individuals that, similarly to antibodies against other respiratory infections, such as influenza, are able to cross the placenta via active transplacental antibody transfer.^7^ COVID-19 vaccines given to pregnant persons may protect infants from severe COVID-19 illness via transplacental transfer of maternal IgG.^8^ Few data exist on maternally derived SARS-CoV-2 antibody in infants born prematurely. Our objective was to evaluate anti-S antibody among paired maternal samples and cord samples from preterm and full-term deliveries.

## Methods

### Participants

Participants were recruited as part of an ongoing prospective cohort study on maternal immunizations in low- and high-risk pregnancies between February 1, 2021, and January 31, 2023. In the parent study, maternal and cord blood samples were collected at delivery after participants provided written informed consent. This study was approved by the University of Washington Human Subjects Division. The study followed the Strengthening the Reporting of Observational Studies in Epidemiology (STROBE) reporting guidelines.^9^

Inclusion criteria for analysis were singleton pregnancy, receipt of 2 doses or more of messenger RNA (mRNA)–based COVID-19 vaccines before delivery, availability of paired maternal and cord samples, no history of prior COVID-19 infection, nondetectable antinucleocapsid (anti-N) antibody, no known fetal genetic anomaly, and infant birth weight appropriate for gestational age (>10th percentile) based on Olsen 2010 growth curves.^10,11^ Participants were excluded from the analysis if they received 1 dose or less of a COVID-19 vaccine before delivery or received a non–mRNA-based vaccine.

### Variables

Clinical data were abstracted from the electronic medical record, including linked Washington State Immunization Registry data, and entered into REDCap (Research Electronic Data Capture*)*, version 12.1.1. Gender, race, and ethnicity data were collected to report diversity representation within our study population. Categorization of race and ethnicity followed Centers for Disease Control and Prevention’s National Health Interview Survey race and ethnicity categories.^12^ Participants self-reported race and ethnic identity and gender during the registration process, which was entered into the electronic medical record. Information regarding demographics is collected at the time of registration or admission at University of Washington. Insurance status included public, private, Tricare (eg, military), federal, and other insurance. Body mass index was based on maternal weight at delivery and was calculated as weight in kilograms divided by height in meters squared. Pregestational diabetes included type 1 or 2 diabetes. We designated participants as having preeclampsia if they were diagnosed with preeclampsia with or without severe features, superimposed preeclampsia, or eclampsia according to American College of Obstetricians and Gynecologists’ criteria.^13^ Participants had chronic hypertension if they were diagnosed with hypertension at less than 20 weeks’ gestational age. Participants with conditions such as systemic lupus erythematous or inflammatory bowel disease were designated as having autoimmune or inflammatory conditions. We considered participants taking immunosuppressing medications as those who received long-term corticosteroids, biologics, or other immunosuppressants. Evidence of SARS-CoV-2 infection was defined as a history of a positive COVID-19 test result by antigen or polymerase chain reaction or a positive anti-N serologic test result.

Participants included in this analysis received either 2 doses or 3 or more doses of mRNA COVID-19 vaccines before delivery and may have received between 0 and 4 doses during pregnancy. We calculated time from last vaccine dose to delivery date and gestational age at last vaccine dose in weeks. Complete data were available for gestational age at delivery, maternal and cord anti-S IgG, number of vaccine doses, timing of doses, and all covariates chosen for adjustment. The missingness in all other variables was less than 1%. No participants were lost to follow-up.

### Antibody Testing

We tested paired maternal and cord samples collected at delivery for anti-S and anti-N antibody using Elecsys Anti-SARS-CoV-2 immunoassays (Roche Diagnostics) at the University of Washington virology laboratory.^14^ This immunoassay is 99.5% sensitive and 99.8% specific for qualitative SARS-CoV-2 anti-N detection.^14^ Results from the immunoassay for anti-S, a semiquantitative assay, and anti-N, a qualitative assay, were converted to geometric mean concentration (GMC) for final analysis.^15^

### Statistical Analysis

Baseline characteristics are reported as absolute numbers and percentages or medians and IQRs. Comparison of baseline characteristics was performed using 2-tailed, unpaired *t* tests, χ^2^ tests, and Fisher exact tests for subgroups with small numbers. We evaluated the association between preterm birth and maternal and cord anti-S antibody levels using linear regression analyses. We conducted similar analyses for cord anti-S antibody levels and cord to maternal anti-S antibody ratios, calculated from untransformed values of maternal and cord anti-S antibody levels. Cord to maternal antibody ratios of 1 or greater are generally considered to indicate efficient transplacental antibody transfer.^16^ The cord to maternal anti-S antibody ratio was normally distributed and compared across the preterm group with 2-tailed, unpaired *t* tests. Anti-S antibody levels were log_2_ transformed and reported as GMCs with 95% CIs. Covariates were chosen a priori and based on significant associations with the exposure of preterm birth and the outcomes of maternal and cord anti-S antibody. Minimally adjusted linear regression models included time between last dose and delivery (in weeks) and number of doses during pregnancy. Fully adjusted models additionally included insurance (private or other) and maternal use of immunosuppressing medications. A sensitivity analysis was completed with the inclusion of gestational age at last vaccination dose to the linear regression models. The first model included time between last dose and delivery, gestational age at last dose, and number of doses. The fully adjusted model additionally included insurance status and immunocompromising medications. Anti-S antibody levels, stratified by the number of vaccine doses before delivery, were also compared in full-term and preterm deliveries using the Wilcoxon rank sum tests for maternal and cord anti-S antibody and 2-tailed, unpaired *t* tests for cord to maternal antibody ratios. A sensitivity analysis was completed to examine maternal, cord, and cord to maternal anti-S antibody ratio between full-term and preterm deliveries based on the number of vaccine doses given during pregnancy. Wilcoxon rank sum tests and 2-tailed, unpaired *t* tests were used for the comparison of anti-S antibody levels. Statistical analysis was performed using Stata software, version 18.0 (StataCorp LLC).^17^ A 2-sided *P* < .05 was considered statistically significant.

## Results

### Baseline Characteristics

A total of 220 participants met the inclusion criteria and had a median age of 34 years (IQR, 32-37 years), with a median gravidity of 2 (IQR, 1-3) and parity of 0 (IQR, 0-1). A total of 212 participants (96.4%) in our study identified as female, 1 (0.5%) identified as other gender, and 7 (3.2%) declined to answer. One participant (0.5%) identified as American Indian or Alaska Native, 26 (11.8%) as Asian, 5 (2.3%) as Black or African American, and 180 (81.8%) as White, with 8 (3.6%) declining to answer; 14 (6.4%) identified as Hispanic and 203 (92.3%) as non-Hispanic, with 3 (1.4%) declining to answer. Most participants (205 [93.2%]) had private insurance. There were 36 and 184 preterm and full-term deliveries, respectively. There were no differences in racial or ethnic identity or insurance status between participants with preterm or full-term delivery (Table 1).

**Table 1.** Demographic and Baseline Characteristics of the Study Participants^a^

Characteristic	Total (N = 220)	Preterm (n = 36)	Full term (n = 184)	*P* value
Enrollment year				
2021	151 (68.6)	10 (27.8)	141 (76.6)	<.001
2022	65 (29.6)	26 (72.2)	39 (21.2)	
2023	4 (1.8)	0	4 (2.2)	
Maternal age, median (IQR), y	34 (32-37)	35 (31-38)	34 (32-36)	.73
Maternal gender				
Female	212 (96.4)	36 (100)	176 (95.7)	<.001
Other	1 (0.5)	0	1 (0.5)	
Unknown or declined to answer	7 (3.2)	0	7 (3.8)	
Gravidity, median (IQR)	2 (1-3)	2 (1-4)	2 (1-3)	.003
Parity, median (IQR)	0 (0-1)	0 (0-1)	0 (0-1)	.08
Race				
American Indian or Alaska Native	1 (0.5)	0	1 (0.5)	.78
Asian	26 (11.8)	3 (8.3)	23 (12.5)	
Black or African American	5 (2.3)	1 (2.8)	4 (2.2)	
White	180 (81.8)	30 (83.3)	150 (81.5)	
Declined to answer	8 (3.6)	2 (5.6)	6 (3.3)	
Ethnicity				
Hispanic	14 (6.4)	5 (13.9)	9 (4.9)	.06
Non-Hispanic	203 (92.3)	31 (86.1)	172 (93.5)	
Declined to answer	3 (1.4)	0	3 (1.6)	
Insurance status				
Public	13 (5.9)	5 (13.9)	8 (4.4)	.04
Private	205 (93.2)	30 (83.3)	175 (95.1)	
Tricare, federal, or other	2 (0.9)	1 (2.8)	1 (0.5)	
BMI, median (IQR)	30.0 (27.2-34.0)	31.8 (28.6-39.0)	29.7 (27.1-33.2)	.02
Pregestational diabetes	8 (3.6)	5 (13.9)	3 (1.6)	.004
Preeclampsia	27 (12.3)	15 (41.7)	12 (6.5)	<.001
Chronic hypertension	19 (8.6)	7 (19.4)	12 (6.5)	.02
Autoimmune or inflammatory disorder	21 (9.6)	3 (8.3)	18 (9.8)	.54
Immunosuppressing medications	14 (6.4)	2 (5.6)	12 (6.5)	.51
No. of COVID-10 vaccine doses before delivery				
2	121 (55.0)	11 (30.6)	110 (59.8)	.006
3	81 (36.8)	21 (58.3)	60 (32.6)	
4	15 (6.8)	4 (11.1)	11 (6.0)	
5	3 (1.4)	0	3 (1.6)	
No. of COVID-10 vaccine doses during pregnancy				
0	19 (8.6)	8 (22.2)	11 (6.0)	<.001
1	56 (25.5)	19 (52.8)	37 (20.1)	
2	121 (55.0)	7 (19.4)	114 (62.0)	
3	22 (10.0)	1 (2.8)	21 (11.4)	
4	2 (0.9)	1 (2.8)	1 (0.5)	
Last vaccine to delivery, median (IQR), wk	16 (7-24)	11 (8-27)	16 (7-24)	.53
Gestational age at last vaccine dose, median (IQR), wk	25 (18-32)	25 (19-29)	25 (18-32)	.54
Maternal total IgG, median (IQR)	682 (565-810)	666 (557-760)	685 (566-821)	.25
Gestational age at delivery, median (IQR) [range], wk	39.3 (37.6-40.2) [27.9-41.9]	35.1 (34.1-36.3) [27.9-36.9]	39.5 (38.7-40.3) [37.0-41.9]	<.001
Mode of delivery				
Vaginal	132 (60.0)	12 (33.3)	120 (65.2)	<.001
Cesarean	88 (40.0)	24 (66.7)	64 (34.8)	
Birth weight, median (IQR), g	3311 (2989-3632)	2437 (2049-2712)	3453 (3180-3713)	<.001
Infant sex				
Female	117 (53.2)	19 (52.8)	98 (53.3)	.96
Male	103 (46.8)	17 (47.2)	86 (46.7)	
NICU admission	33 (15.0)	23 (63.9)	10 (5.4)	<.001

Abbreviations: BMI, body mass index (calculated as weight in kilograms divided by height in meters squared); NICU, neonatal intensive care unit.

^a^ Data are presented as number (percentage) unless otherwise indicated. Continuous variables were compared using a 2-tailed, unpaired *t* test, and categorical variables were compared using the χ^2^ test and Fisher exact test for variables with subgroup sample sizes below 5.

Participants with preterm delivery had a significantly higher median body mass index (preterm: 31.8 [IQR, 28.6-39.0]; full term: 29.7 [IQR, 27.1-33.2]; *P* = .02), higher rates of pregestational diabetes (preterm: 5 [13.9%]; full term: 3 [1.6%]; *P* < .004), preeclampsia (preterm: 15 [41.7%]; full term: 12 [6.5%]; *P* < .001), and chronic hypertension (preterm: 7 [19.4%]; full term: 12 [6.5%]; *P* = .02). There was no difference in autoimmune or inflammatory disease, receipt of immunosuppressing medications, or maternal total IgG concentrations between groups (Table 1).

### Pregnancy Outcomes

Preterm infants were delivered at a median gestational age of 35.1 weeks (IQR, 34.1-36.3 weeks), ranging from 27.9 to 36.9 weeks, whereas full-term infants were delivered at a median of 39.5 weeks (IQR, 38.7-40.3 weeks) (*P* < .001) (Table 1). Of preterm deliveries, 7 (19.4%) delivered at less than 34 weeks’ gestational age. Pregnancies with preterm delivery were more likely to be delivered via cesarean section (preterm: 24 [66.7%]; full term: 64 [34.8%]; *P* < .001), have lower median birth weight (preterm: 2437 g [IQR, 2049-2712 g]; full-term: 3453 g [IQR, 3180-3713]; *P* < .001), and be admitted to the neonatal intensive care unit (preterm: 23 [63.9%]; full term: 10 [5.4%]; *P* < .001).

### Vaccine History

Before delivery, 121 (55.0%) and 99 (45.0%) persons received 2 and 3 or more COVID-19 vaccine doses at any time (ie, before or during pregnancy), respectively. More participants with preterm delivery received 3 or more doses before delivery (preterm: 25 [69.4%]; full term: 74 [40.2%]) compared with 2 doses (preterm: 11 [30.6%]; full term: 110 [59.8%]; *P* = .006). The number of vaccine doses during pregnancy also significantly differed between pregnancies with preterm and full-term deliveries, with 7 patients (19.4%) with preterm delivery receiving 2 doses compared with 114 participants (62.0%) with full-term delivery (*P* < .001). Median time between last vaccine dose and delivery was 16 weeks (IQR, 7-24 weeks); median gestational age at last dose was 25 weeks (IQR, 18-32 weeks), without difference between preterm and full-term pregnancies (Table 1).

### Anti-S Antibody Analyses

**Figure 1.**
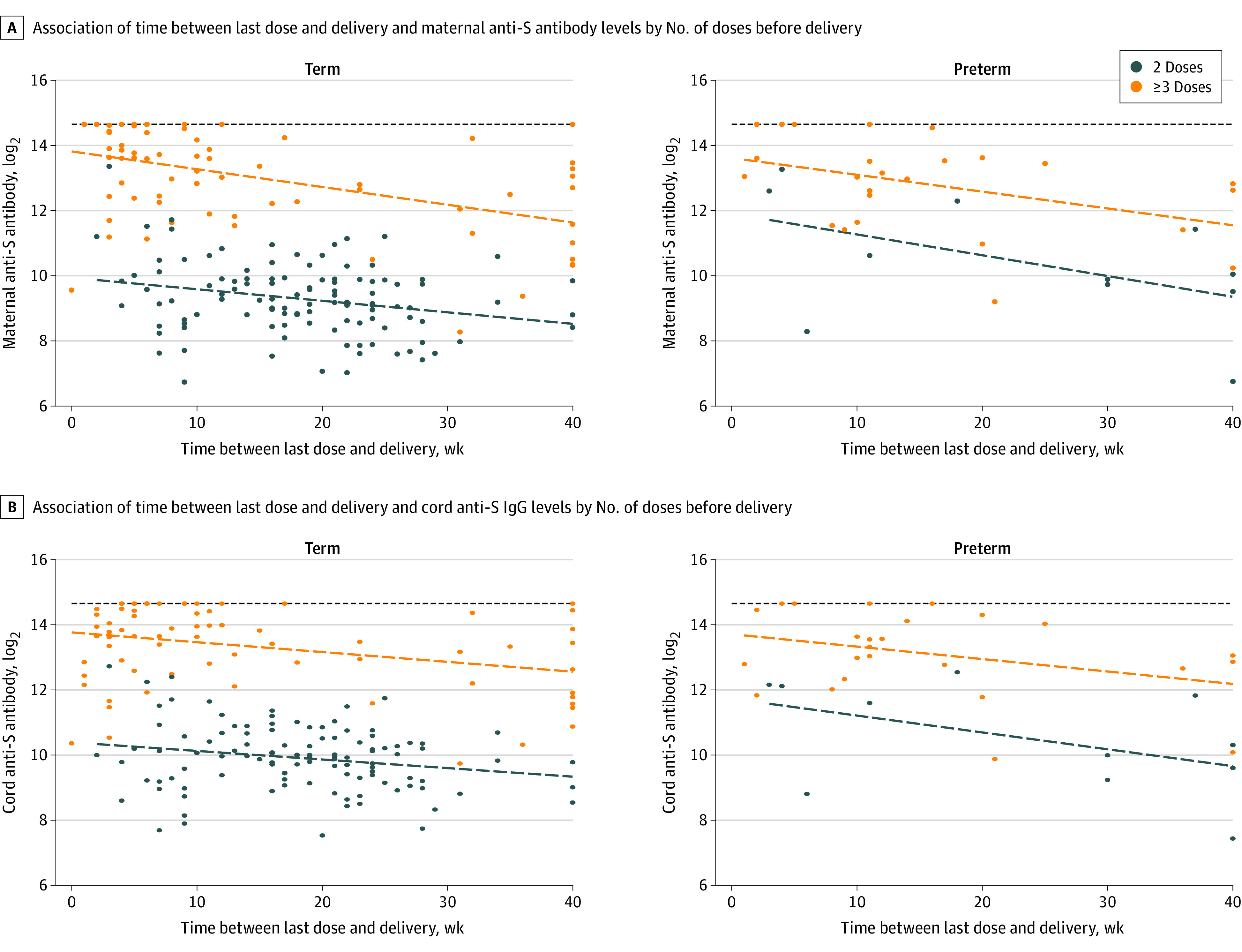
Maternal and Cord Anti-Spike (Anti-S) Protein Antibody Concentrations by Time From Last Vaccine Dose to Delivery A, Scatterplot and linear fit line for the relationship between time between last dose and delivery and maternal anti-S IgG levels by number of doses prior to delivery, stratified by preterm status. B, Scatterplot and linear fit line for the relationship between time between last dose and delivery and cord anti-S IgG levels by number of doses prior to delivery, stratified by preterm status. The horizontal dashed lines are the upper testing limit of the assay.

**Figure 2.**
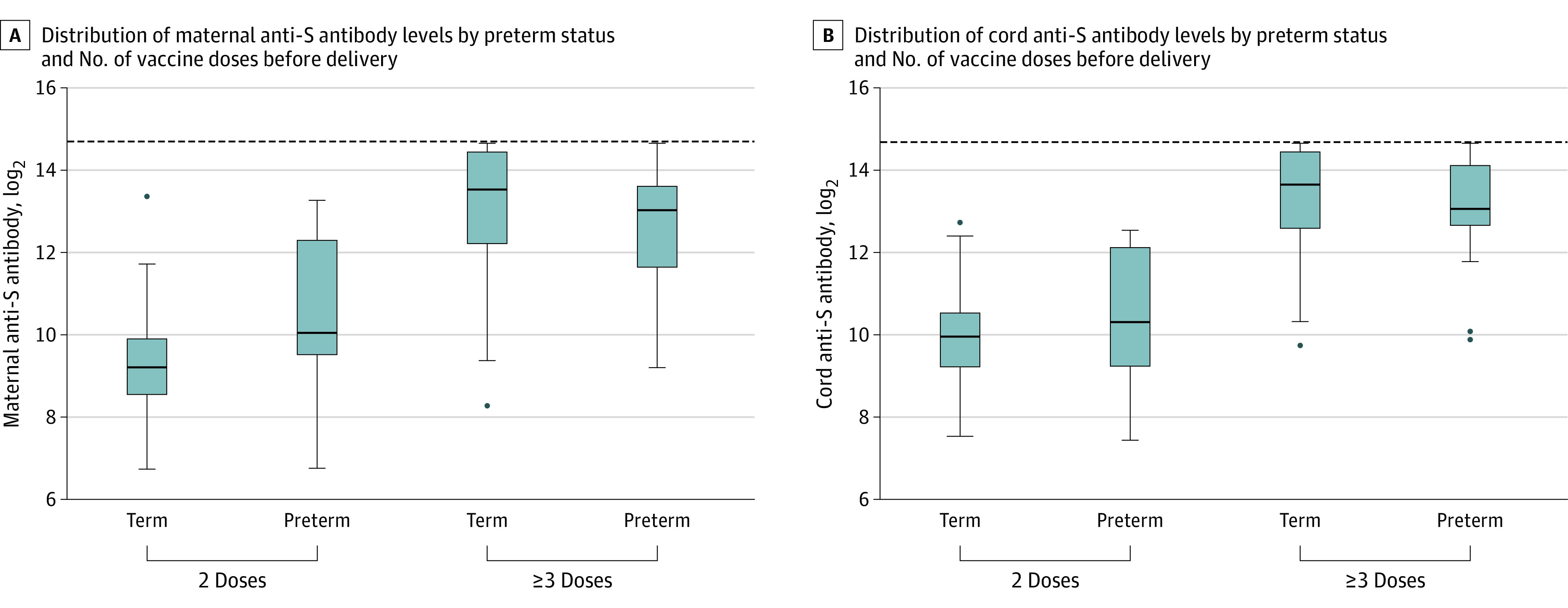
Maternal and Cord Anti-Spike (Anti-S) Protein IgG Concentrations by Number of COVID-19 Doses Received Before Delivery The horizontal lines in the boxes are the medians; upper and lower ends of the boxes indicate the 25th and 75th percentiles. Whiskers indicate all data points within 1.5 IQR of the upper and lower quartiles. Dots indicate outliers. The horizontal dashed lines indicate the upper testing limit of the assay.

After 2 doses, the unadjusted maternal anti-S antibody GMC was 674 (95% CI, 577-787), and after 3 or more doses, the GMC was 8159 (95% CI, 6636-10 032) (*P* < .001). Unadjusted cord anti-S antibody GMC was 1000 (95% CI, 874-1144) and 9992 (95% CI, 8381-11 914) after 2 and 3 or more doses, respectively (*P* < .001) (Table 2, Figure 1, and Figure 2). Overall, pregnancies with preterm deliveries had lower cord to maternal antibody ratios (preterm: 1.18 [95% CI, 1.02-1.38]; full term: 1.40 [95% CI, 1.31-1.49]; *P* = .02) (Table 2). Pregnancies delivered at less than 34 weeks’ gestational age had cord to maternal antibody ratios of 0.97 (95% CI, 0.57-1.66) compared with those delivered late preterm (ratio, 1.24; 95% CI, 1.06-1.45) and full term (ratio, 1.40; 95% CI, 1.31-1.49) (*P* = .34) (eTable 1 in Supplement 1).

**Table 2.** Maternal and Cord Anti-S Antibody Levels^a^

Variable	Geometric mean concentration (95% CI)			*P* value
	Total	Preterm	Full term	
Overall				
No. of participants	220	36	184	NA
Maternal spike	2070 (1682-2547)	4299 (2733-6762)	1794 (1429-2252)	.002
Cord spike	2817 (2338-3395)	5090 (3300-7850)	2509 (2046-3076)	.007
Ratio	1.36 (1.28-1.44)	1.18 (1.02-1.38)	1.40 (1.31-1.49)	.02
2 Doses				
No. of participants	121	11	110	NA
Maternal spike	674 (577-787)	1356 (553-3326)	628 (543-727)	.03
Cord spike	1000 (874-1144)	1460 (675-3157)	963 (845-1097)	.20
Ratio	1.48 (1.39-1.58)	1.08 (0.81-1.42)	1.53 (1.44-1.63)	.005
≥3 Doses				
No. of participants	99	25	74	NA
Maternal spike	8159 (6636-10 032)	7141 (4733-10 774)	8535 (6689-10 890)	.32
Cord spike	9992 (8381-11 914)	8818 (6078-12 793)	10 423 (8506-12 773)	.34
Ratio	1.22 (1.10-1.36)	1.23 (1.02-1.50)	1.22 (1.08-1.38)	.87

Abbreviations: anti-S, anti-Spike; NA, not applicable.

^a^ Maternal and cord anti-S antibody were compared using Wilcoxon rank sum test for nonnormal continuous variables; the ratio is calculated from untransformed values as cord anti-S antibody level divided by maternal anti-S antibody level and compared using a 2-tailed, unpaired *t* test for normally distributed continuous variables.

We ran 2 separate linear regression analyses to adjust for vaccine dose timing and other covariates found to be associated with anti-S IgG concentration. In our first model, we adjusted for the number of COVID-19 vaccine doses during pregnancy and time between last vaccine dose and delivery. After adjustment in our first model, there was no association between preterm delivery and cord anti-S antibody levels (β = 0.46; 95% CI, −0.03 to 0.95). In our second model, we additionally adjusted for insurance status and immunosuppressing medication use. In this model, no significant difference between cord anti-S antibody levels was found between pregnancies with preterm and full-term deliveries (β = 0.44; 95% CI, −0.06 to 0.94). Maternal anti-S antibody levels were significantly higher and cord to maternal anti-S antibody ratios significantly lower in pregnancies with preterm deliveries compared with those with full-term deliveries (Table 3).

**Table 3.** Linear Regression Analyses of the Association Between Preterm Birth and Maternal and Cord Anti-S Antibody Levels, Adjusted for Timing of Last Dose and Number of Doses Before Delivery

Birth	Overall		Model 1^a^		Model 2^b^	
	β Coefficient (95% CI)	*P* value	Adjusted β coefficient (95% CI)	*P* value	Adjusted β coefficient (95% CI)	*P* value
**Maternal anti-S antibody**						
Full term	0 [Reference]	NA	0 [Reference]	NA	0 [Reference]	NA
Preterm	1.26 (0.47 to 2.05)	.002	0.68 (0.13 to 1.23)	.02	0.66 (0.10 to 1.22)	.02
**Cord anti-S antibody**						
Full term	0 [Reference]	NA	0 [Reference]	NA	0 [Reference]	NA
Preterm	1.02 (0.30 to 1.74)	.005	0.46 (−0.03 to 0.95)	.07	0.44 (−0.06 to 0.94)	.08
**Cord to maternal anti-S antibody ratio **						
Full term	0 [Reference]	NA	0 [Reference]	NA	0 [Reference]	NA
Preterm	−0.22 (−0.42 to −0.03)	.02	−0.21 (−0.40 to −0.02)	.04	−0.20 (−0.40 to −0.004)	.04

Abbreviations: anti-S, anti-Spike; NA, not applicable.

^a^ Model 1 was adjusted for time between last dose and delivery in weeks and number of doses before delivery.

^b^ Model 2 was adjusted for time between last dose and delivery in weeks, number of doses before delivery, insurance (private or other), and immunocompromising drugs (yes or no).

We also ran a linear regression analysis to adjust for gestational age at last vaccine dose and other covariates associated with anti-S antibody concentration. For this analysis, 19 participants were excluded (8 with preterm deliveries and 11 with full-term deliveries) due to no vaccine dose during pregnancy. After adjustment in our first model, there was no significant association between preterm delivery and cord anti-S titers (β = 0.28; 95% CI, −0.31 to 0.87) (eTable 2 in Supplement 1). In our second model, additionally adjusted for insurance status and immunosuppressing medication use, there was also no significant difference between cord anti-S concentrations in pregnancies with preterm and full-term deliveries (β = 0.26; 95% CI, −0.34 to 0.86). Maternal anti-S antibody concentrations and cord to maternal anti-S antibody ratios were similar in pregnancies with preterm compared with full-term deliveries (eTable 2 in Supplement 1). In our sensitivity analysis examining anti-S antibody levels stratified by number of doses during pregnancy, maternal and cord anti-S antibody and cord to maternal ratios did not differ between preterm and full-term deliveries in pregnancies with 0 to 1 vaccine doses. In participants with 2 doses during pregnancy, maternal anti-S antibody levels, cord anti-S antibody levels, and cord to maternal antibody ratios differed between preterm and full-term deliveries (eTable 3 in Supplement 1). Cord to maternal antibody ratios peaked at approximately 10 weeks after last vaccine dose (eFigure in Supplement 1).

## Discussion

In this cohort study of COVID-19 vaccine among pregnancies with preterm and full-term deliveries, we found that, when adjusted for vaccine dose timing before delivery, cord anti-S antibody concentrations were similar in pregnancies with preterm compared with full-term deliveries. Furthermore, participants who had 3 or more doses of mRNA-based vaccines before delivery had significantly higher concentrations of maternal anti-S antibodies, resulting in 10-fold higher cord anti-S antibody levels. The finding that 3 or more doses of COVID-19 vaccine significantly enhance antibody concentrations compared with 2 doses has been reported in other observational pregnancy studies,^7,18^ however, not in the comparison of preterm vs full-term infants. In nonpregnant adults, 3 or more doses of COVID-19 vaccine were less likely to be associated with symptomatic COVID-19 infection; this may be important for the pregnant population given the increased morbidity and mortality associated with COVID-19 illness in pregnancy.^19^ Regarding potential infant protection, follow-up studies on infant morbidity with COVID-19 illness have been conducted among pregnant persons who received COVID-19 vaccine^8,20^; however, these studies only evaluated pregnancies with 2 vaccine doses. Additional studies are currently being conducted to evaluate clinical outcomes among pregnant individuals and their infants after 3 COVID-19 vaccine doses. There is currently no clearly recognized immunologic correlate of protection for SARS-CoV-2, and transfer properties of antibodies generated by COVID-19 vaccines and SARS-CoV-2 antibodies in general may not be generalizable to other vaccines and other pathogen-specific antibodies. However, on the basis of the significantly enhanced antibody response after 3 or more vaccine doses, pregnant individuals should be encouraged to receive a booster before delivery. Individuals without known COVID-19 infection should be encouraged to receive the full COVID-19 vaccine series.

Maternally derived IgG antibody can be beneficial for premature infants given their higher risk for infection and adverse outcomes with infections in early life. Early studies on transplacental antibody transfer have shown that concentrations of maternal IgG increase steadily during gestation and that placental efficiency in transplacental IgG transfer is highly dependent on gestational age.^16,21,22^ Concentrations of circulating fetal IgG increase from approximately 10% of the maternal concentration at 17 to 22 weeks to 50% at 28 to 32 weeks to greater than 100% of maternal antibody concentration at full term.^16,23^ A cross-sectional study of 213 maternal-infant pairs by Okoko et al^24^ from 2001 demonstrated lower antibody titers and transfer ratios in cord blood of infants born prematurely compared with full-term infants, but this was confounded by maternal malaria and uncertain timing of pathogen or vaccine exposure. More recently in 2014, van den Berg et al^25^ found that cord to maternal IgG ratios for measles, mumps, rubella, and varicellaispecific antigens were significantly lower for preterm compared with full-term infants but that in preterm infants the influence of gestational age on maternally derived IgG levels in cord blood was less strong compared with the impact of maternal IgG concentrations.^25^ Consistent with our findings, van den Berg et al^25^ found that in preterm infants, the influence of gestational age on maternally derived IgG levels in cord blood was less strong compared with the impact of maternal IgG concentrations.^25^ Although placental transfer ratios may be helpful in the context of gestational age to project potential concentrations of maternal IgG in the infant, our findings suggest that we may be able to improve potential infant immune protection by changing the framework of the discussion from transfer ratio to ultimate final concentration of maternally derived IgG in cord blood. If the focus is on maternal IgG titers in cord samples, this study and prior studies would suggest that pathogen-specific IgG concentrations in maternal blood must first be optimized to increase infant IgG concentrations. If maternal antibody concentrations are not boosted until the third trimester, many infants born prematurely will benefit less from maternal immunization.

The timing of vaccination during pregnancy is vital to maximize maternal antibody response as well as protection from severe COVID-19 illness and transplacental antibody transfer. Prior studies suggest waning maternal and cord anti-S antibody levels over time. In a prospective cohort study of 178 mother-infant pairs, Zilver et al^26^ observed lower cord IgG levels at birth with greater time between last vaccine dose and delivery. Similarly, waning antibody concentrations were seen by Atyeo at al^27^ in their prospective study of 123 maternal-neonatal dyads. Additional studies^28,29^ found specifically that early third trimester vaccination results in higher IgG ratios, suggesting a buffer period before delivery is needed to boost maternal IgG concentrations and allow time for transplacental antibody transfer. Our data are consistent with prior studies showing a cord to maternal IgG ratio peak at approximately 10 weeks after vaccine dose. It is important to consider risk of waning maternal IgG concentrations while also emphasizing maternal protection from COVID-19 as well as targeting premature infants. Timing considerations for COVID-19 vaccine administration during pregnancy should consider individuals at risk for preterm delivery where mortality and morbidity are high.

### Strengths and Limitations

This study has several strengths. We derived our study population from an institution with large numbers of both low- and high-risk pregnancies and selected our study participants based on stringent criteria, including receipt of mRNA-based vaccines only and no history or serologic evidence of prior COVID-19 infection based on negative test results for anti-N antibody. Hence, the concentrations of infant anti-S IgG in our study are derived solely from maternal immunization and not from prior COVID-19 infection. In addition, the Washington State Vaccination Registry provided the ability to obtain accurate vaccine data on our participants.

This study also has some limitations. Although our study is one of the largest on anti-S concentrations and vaccine timing in preterm and full-term deliveries, we were still limited by sample size and preterm deliveries that were confined to the third trimester (range, 27.9-36.9 weeks’ gestational age), and additional confounders may be present that we were not able to adjust for. Our selection criteria for this analysis based on at least 2 doses of mRNA-based vaccines before delivery may limit our population studied, as a prior study^29^ found differences in COVID-19 vaccine acceptance among racial and ethnic groups. We also do not report results from neutralizing assays; however, a previous study^30^ found a good correlation between anti-S antibody levels and neutralizing function. In addition, the Elecsys immunoassay has an upper limit of 25 720 BAU/mL, which was reached by 19 maternal and 19 cord samples in this study. The immunoassay also tests predominantly for IgG but may pick up small quantities of IgA and IgM.^12^ Although characteristics of this immunoassay do not affect analyses of maternal and cord antibody concentrations, they may limit interpretation of cord to maternal antibody ratios.

## Conclusions

Our data suggest that receipt of 2 or fewer COVID-19 vaccine doses may not provide optimal SARS-CoV-2 antibody titers and therefore protection for pregnant individuals and may not enable sufficient antibody transfer via cord blood for optimal infant protection, particularly in pregnant individuals who did not receive a primary COVID-19 vaccine series. As we note, our study excluded individuals with a history or serologic evidence of a prior COVID-19 infection, so we are not able to evaluate the impact of natural immunity from SARS-CoV-2 infection on infant antibody levels, and we acknowledge that an increasing proportion of the population has become infected with SARS-CoV-2. However, with the current primary COVID-19 vaccine series no longer available and a recent Advisory Committee on Immunization Practices recommendation for a single vaccine dose for all individuals, further study is warranted regarding COVID-19 vaccine dosing to provide optimal maternal and infant COVID-19 antibody protection. Finally, this study demonstrates that maternal anti-S antibody concentration is an important determinant of cord anti-S antibody levels. In individuals at risk for preterm delivery, timing of COVID-19 vaccine administration merits consideration.

## Notes

Supplement 1. **eTable 1.** Maternal and Cord Anti-Spike (S) Antibody Levels by Early Preterm, Late Preterm, and Term **eTable 2.** Linear Regression Analyses of the Association Between Preterm Birth and Maternal and Cord Anti-Spike (S) Antibody Levels, Adjusted for Gestational Age at Last Dose, Time Between Last Dose and Delivery, and Number of Doses Prior to Delivery **eTable 3.** Maternal and Cord Anti-Spike (S) Antibody Levels by Number of Doses During Pregnancy **eFigure**. Scatterplot and Lowess Smoothing Line for the Relationship Between Time Between Last Dose and Delivery and Cord:Maternal Anti-Spike (S) Antibody Ratios by Preterm Status, Stratified by Number of Doses

Supplement 2.
Data Sharing Statement

## References

[1] Stock SJ, Carruthers J, Calvert C, . SARS-CoV-2 infection and COVID-19 vaccination rates in pregnant women in Scotland. Nat Med. 2022;28(3):504-512. doi:10.1038/s41591-021-01666-2 35027756 PMC8938271

[2] Goldshtein I, Nevo D, Steinberg DM, . Association between BNT162b2 vaccination and incidence of SARS-CoV-2 infection in pregnant women. JAMA. 2021;326(8):728-735. doi:10.1001/jama.2021.11035 34251417 PMC8276131

[3] Dagan N, Barda N, Biron-Shental T, . Effectiveness of the BNT162b2 mRNA COVID-19 vaccine in pregnancy. Nat Med. 2021;27(10):1693-1695. doi:10.1038/s41591-021-01490-8 34493859

[4] Delahoy MJ, Whitaker M, O’Halloran A, ; COVID-NET Surveillance Team. Characteristics and maternal and birth outcomes of hospitalized pregnant women with laboratory-confirmed COVID-19—COVID-NET, 13 states, March 1-August 22, 2020. MMWR Morb Mortal Wkly Rep. 2020;69(38):1347-1354. doi:10.15585/mmwr.mm6938e1 32970655 PMC7727497

[5] Zambrano LD, Ellington S, Strid P, ; CDC COVID-19 Response Pregnancy and Infant Linked Outcomes Team. Update: characteristics of symptomatic women of reproductive age with laboratory-confirmed SARS-CoV-2 infection by pregnancy status—United States, January 22-October 3, 2020. MMWR Morb Mortal Wkly Rep. 2020;69(44):1641-1647. doi:10.15585/mmwr.mm6944e3 33151921 PMC7643892

[6] Villar J, Soto Conti CP, Gunier RB, ; INTERCOVID-2022 International Consortium. Pregnancy outcomes and vaccine effectiveness during the period of omicron as the variant of concern, INTERCOVID-2022: a multinational, observational study. Lancet. 2023;401(10375):447-457. doi:10.1016/S0140-6736(22)02467-9 36669520 PMC9910845

[7] Yang YJ, Murphy EA, Singh S, . Association of gestational age at coronavirus disease 2019 (COVID-19) vaccination, history of severe acute respiratory syndrome coronavirus 2 (SARS-CoV-2) infection, and a vaccine booster dose with maternal and umbilical cord antibody levels at delivery. Obstet Gynecol. 2022;139(3):373-380. doi:10.1097/AOG.0000000000004693 34963127

[8] Halasa NB, Olson SM, Staat MA, ; Overcoming COVID-19 Investigators; Overcoming COVID-19 Network. Effectiveness of maternal vaccination with mRNA COVID-19 vaccine during pregnancy against COVID-19-associated hospitalization in infants aged <6 months—17 states, July 2021-January 2022. MMWR Morb Mortal Wkly Rep. 2022;71(7):264-270. doi:10.15585/mmwr.mm7107e3 35176002 PMC8853480

[9] Equator Network. The Strengthening the Reporting of Observational Studies in Epidemiology (STROBE) Statement: guidelines for reporting observational studies: Equator Network; 2021. Updated September 10, 2021. Accessed December 6, 2023. https://www.equator-network.org/reporting-guidelines/strobe/

[10] Olsen IE, Groveman SA, Lawson ML, Clark RH, Zemel BS. New intrauterine growth curves based on United States data. Pediatrics. 2010;125(2):e214-e224. doi:10.1542/peds.2009-0913 20100760

[11] Ferguson AN, Olsen IE, Clark RH, . Differential classification of infants in United States neonatal intensive care units for weight, length, and head circumference by United States and international growth curves. Ann Hum Biol. 2020;47(6):564-571. doi:10.1080/03014460.2020.1817555 32945183

[12] Centers for Disease Control and Prevention. National Health Interview Survey: glossary. Accessed December 6, 2023. https://www.cdc.gov/nchs/nhis/rhoi/rhoi_glossary.htm

[13] Gestational hypertension and preeclampsia: ACOG practice bulletin summary, number 222. Obstet Gynecol. 2020;135(6):1492-1495. doi:10.1097/AOG.0000000000003892 32443077

[14] Roche Diagnostics GmbH. Elecsys® Anti-SARS-CoV-2. Accessed December 6, 2023. https://diagnostics.roche.com/us/en/products/params/elecsys-anti-sars-cov-2.html

[15] Roche Diagnostics Department of Research & Development. Correlation of the units (U) of the Elecsys® Anti-SARS-CoV-2 S assay to the “binding antibody units” (BAU) of the first WHO International Standard for anti-SARS-CoV-2 immunoglobulin. Roche Diagnostics; 2021:12.

[16] Malek A, Sager R, Kuhn P, Nicolaides KH, Schneider H. Evolution of maternofetal transport of immunoglobulins during human pregnancy. Am J Reprod Immunol. 1996;36(5):248-255. doi:10.1111/j.1600-0897.1996.tb00172.x 8955500

[17] StataCorp. Stata Statistical Software: Release 17. StataCorp LLC; 2021.

[18] Kugelman N, Nahshon C, Shaked-Mishan P, . Maternal and neonatal severe acute respiratory syndrome coronavirus 2 (SARS-CoV-2) immunoglobulin G levels after the Pfizer-BioNTech booster dose for coronavirus disease 2019 (COVID-19) vaccination during the second trimester of pregnancy. Obstet Gynecol. 2022;140(2):187-193. doi:10.1097/AOG.0000000000004867 35852268

[19] Accorsi EK, Britton A, Fleming-Dutra KE, . Association between 3 doses of mRNA COVID-19 vaccine and symptomatic infection caused by the SARS-CoV-2 Omicron and Delta variants. JAMA. 2022;327(7):639-651. doi:10.1001/jama.2022.0470 35060999 PMC8848203

[20] Zerbo O, Ray GT, Fireman B, . Maternal SARS-CoV-2 vaccination and infant protection against SARS-CoV-2 during the first six months of life. Nat Commun. 2023;14(1):894. doi:10.1038/s41467-023-36547-4 36854660 PMC9974935

[21] Kohler PF, Farr RS. Elevation of cord over maternal IgG immunoglobulin: evidence for an active placental IgG transport. Nature. 1966;210(5040):1070-1071. doi:10.1038/2101070a0 5950290

[22] Kachikis A, Englund JA. Maternal immunization: optimizing protection for the mother and infant. J Infect. 2016;72(suppl):S83-S90. doi:10.1016/j.jinf.2016.04.027 27233120

[23] van den Berg JP, Westerbeek EA, van der Klis FR, Berbers GA, van Elburg RM. Transplacental transport of IgG antibodies to preterm infants: a review of the literature. Early Hum Dev. 2011;87(2):67-72. doi:10.1016/j.earlhumdev.2010.11.003 21123010

[24] Okoko BJ, Wesuperuma LH, Ota MO, . Influence of placental malaria infection and maternal hypergammaglobulinaemia on materno-foetal transfer of measles and tetanus antibodies in a rural west African population. J Health Popul Nutr. 2001;19(2):59-65. 11503348

[25] van den Berg JP, Westerbeek EA, Smits GP, van der Klis FR, Berbers GA, van Elburg RM. Lower transplacental antibody transport for measles, mumps, rubella and varicella zoster in very preterm infants. PLoS One. 2014;9(4):e94714. doi:10.1371/journal.pone.0094714 24728480 PMC3984210

[26] Zilver SJM, de Groot CJM, Grobben M, . Vaccination from the early second trimester onwards gives a robust SARS-CoV-2 antibody response throughout pregnancy and provides antibodies for the neonate. Int J Infect Dis. 2023;130:126-135. doi:10.1016/j.ijid.2023.02.022 36868302 PMC9977072

[27] Atyeo CG, Shook LL, Brigida S, . Maternal immune response and placental antibody transfer after COVID-19 vaccination across trimester and platforms. Nat Commun. 2022;13(1):3571. doi:10.1038/s41467-022-31169-8 35764643 PMC9239994

[28] Prahl M, Golan Y, Cassidy AG, . Evaluation of transplacental transfer of mRNA vaccine products and functional antibodies during pregnancy and infancy. Nat Commun. 2022;13(1):4422. doi:10.1038/s41467-022-32188-1 35908075 PMC9338928

[29] Rottenstreich A, Zarbiv G, Oiknine-Djian E, . Timing of SARS-CoV-2 vaccination during the third trimester of pregnancy and transplacental antibody transfer: a prospective cohort study. Clin Microbiol Infect. 2022;28(3):419-425. doi:10.1016/j.cmi.2021.10.003 34740773 PMC8563509

[30] Roche Diagnostics GmbH. Elecsys Anti-SARS-CoV-2 S: instructions for use. September 25, 2023. Accessed December 6, 2023. https://www.fda.gov/media/144037/download

