# Supplementary material for: Timing of Maternal COVID-19 Vaccine and Antibody Concentrations in Infants Born Preterm
Source: JAMA Netw Open. 2024 Jan 19;7(1):e2352387. doi: 10.1001/jamanetworkopen.2023.52387 (PMC10799259; doi:10.1001/jamanetworkopen.2023.52387)
Supplement: Supplement 1. — eTable 1. Maternal and Cord Anti-Spike (S) Antibody Levels by Early Preterm, Late Preterm, and Term eTable 2. Linear Regression Analyses of the Association Between Preterm Birth and Maternal and Cord Anti-Spike (S) Antibody Levels, Adjusted for Gestational Age at Last Dose, Time Between Last Dose and Delivery, and Number of Doses Prior to Delivery eTable 3. Maternal and Cord Anti-Spike (S) Antibody Levels by Number of Doses During Pregnancy eFigure. Scatterplot and Lowess Smoothing Line for the Relationship Between Time Between Last Dose and Delivery and Cord:Maternal Anti-Spike (S) Antibody Ratios by Preterm Status, Stratified by Number of Doses [file jamanetwopen-e2352387-s001.pdf]

# Supplementary Online Content

Kachikis A, Pike M, Eckert LO, et al. Timing of parental COVID-19 vaccine and antibody concentrations in infants born preterm. *JAMA Netw Open*. 2024;7(1): e2352387. doi:10.1001/jamanetworkopen.2023.52387

**eTable 1.** Maternal and Cord Anti-Spike (S) Antibody Levels by Early Preterm, Late Preterm, and Term
**eTable 2.** Linear Regression Analyses of the Association Between Preterm Birth and Maternal and Cord Anti-Spike (S) Antibody Levels, Adjusted for Gestational Age at Last Dose, Time Between Last Dose and Delivery, and Number of Doses Prior to Delivery
**eTable 3.** Maternal and Cord Anti-Spike (S) Antibody Levels by Number of Doses During Pregnancy
**eFigure.** Scatterplot and Lowess Smoothing Line for the Relationship Between Time Between Last Dose and Delivery and Cord:Maternal Anti-Spike (S) Antibody Ratios by Preterm Status, Stratified by Number of Doses

This supplementary material has been provided by the authors to give readers additional information about their work.

© 2024 Kachikis A et al. *JAMA Network Open.*

**eTable 1.** Maternal and Cord Anti-Spike (S) Antibody Levels by Early Preterm, Late Preterm, and Term

| | Total | Early Preterm <34 weeks GA | Late Preterm ≥34 - <37 weeks GA | Term ≥37 weeks GA | P-value |
|---|---|---|---|---|---|
| **Overall** | **n=220** | **n=7** | **n=29** | **n=184** | |
| Maternal anti-S | 2070 (1682-2547) | 2635 (958-7250) | 4837 (2860-8183) | 1794 (1429-2252) | 0.005 |
| Cord anti-S | 2817 (2338-3395) | 2557 (999-6542) | 6011 (3671-9842) | 2509 (2046-3076) | 0.01 |
| Ratio | 1.36 (1.28-1.44) | 0.97 (0.57-1.66) | 1.24 (1.06-1.45) | 1.40 (1.31-1.49) | 0.34 |

Notes: Geometric mean concentrations (95% confidence intervals); maternal and cord anti-spike antibody levels compared using Kruskal Wallis rank test for non-normal continuous variables; ratio is calculated from untransformed values as cord anti-spike antibody concentration divided by maternal anti-spike antibody concentration and compared using one-way ANOVA for normally distributed continuous variables

Abbreviations: anti-S = anti-Spike antibody; GA = gestational age

© 2024 Kachikis A et al. *JAMA Network Open.*

**eTable 2.** Linear Regression Analyses of the Association Between Preterm Birth and Maternal and Cord Anti-Spike (S) Antibody Levels, Adjusted for Gestational Age at Last Dose, Time Between Last Dose and Delivery, and Number of Doses Prior to Delivery

| | β-coefficient (95% CI) | P-value | *Adjusted β-coefficient (95% CI) | P-value | +Adjusted β-coefficient (95% CI) | P-value |
|---|---|---|---|---|---|---|
| **Maternal Anti-S** | | | | | | |
| Term | 0.0 (ref) | - | 0.0 (ref) | - | 0.0 (ref) | - |
| Preterm | 1.26 (0.47-2.05) | 0.002 | 0.47 (-0.17-1.12) | 0.150 | 0.44 (-0.21-1.10) | 0.182 |
| **Cord Anti-S** | | | | | | |
| Term | 0.0 (ref) | - | 0.0 (ref) | - | 0.0 (ref) | - |
| Preterm | 1.02 (0.30-1.74) | 0.005 | 0.28 (-0.31-0.87) | 0.343 | 0.26 (-0.34-0.86) | 0.397 |
| **Ratio: cord/maternal anti-S** | | | | | | |
| Term | 0.0 (ref) | - | 0.0 (ref) | - | 0.0 (ref) | - |
| Preterm | -0.22 (-0.42 to -0.03) | 0.025 | -0.17 (-0.40-0.06) | 0.139 | -0.17 (-0.40-0.06) | 0.153 |

*Adjusted for GA at last dose, time between last dose and delivery (weeks), and number of doses prior to delivery
+Adjusted for GA at last dose, time between last dose and delivery (weeks), number of doses prior to delivery, insurance (private/other), and immunocompromising drugs (yes/no)
Abbreviations: CI = confidence interval; GA = gestational age; anti-S = anti-Spike antibody

© 2024 Kachikis A et al. *JAMA Network Open.*

**eTable 3.** Maternal and Cord Anti-Spike (S) Antibody Levels by Number of Doses During Pregnancy

| | Total | Preterm | Term | P-value |
|---|---|---|---|---|
| **Overall** | n=220 | n=36 | n=184 | |
| Maternal anti-S | 2070 (1682-2547) | 4299 (2733-6762) | 1794 (1429-2252) | 0.002 |
| Cord anti-S | 2817 (2338-3395) | 5090 (3300-7850) | 2509 (2046-3076) | 0.007 |
| Ratio | 1.36 (1.28-1.44) | 1.18 (1.02-1.38) | 1.40 (1.31-1.49) | 0.025 |
| **0-1 Doses** | n=75 | n=27 | n=48 | |
| Maternal anti-S | 5548 (4162-7396) | 4661 (2832-7669) | 6120 (4262-8788) | 0.26 |
| Cord anti-S | 7232 (5473-9554) | 5731 (3497-9392) | 8242 (5848-11616) | 0.14 |
| Ratio | 1.30 (1.18-1.43) | 1.23 (1.03-1.47) | 1.35 (1.20-1.51) | 0.41 |
| **2 Doses** | n=121 | n=7 | n=114 | |
| Maternal anti-S | 826 (674-1013) | 3237 (803-13051) | 760 (624-925) | 0.011 |
| Cord anti-S | 1248 (1038-1501) | 3315 (997-11026) | 1176 (980-1410) | 0.031 |
| Ratio | 1.51 (1.42-1.61) | 1.02 (0.66-1.59) | 1.55 (1.46-1.64) | 0.011 |
| **3+ Doses** | n=24 | n=2 | n=22 | |
| Maternal anti-S | 9730 (5959-15887) | 3894* | 10574 (6713-16657) | 0.71 |
| Cord anti-S | 8953 (6127-13081) | 4603* | 9511 (6750-13399) | 0.68 |
| Ratio | 0.92 (0.69-1.23) | 1.18* | 0.90 (0.66-1.23) | 0.76 |

Notes: geometric mean concentration (95% confidence interval); maternal and cord anti-S antibody compared using Wilcoxon rank sum test; ratio is calculated as cord anti-spike antibody level divided by maternal anti-spike antibody level and compared using t-test for normally distributed continuous variables.
*95% CIs not computed for participants with preterm birth and 3+ doses during pregnancy due to small numbers.
Abbreviations: anti-S = anti-Spike antibody

**eFigure**. Scatterplot and Lowess Smoothing Line for the Relationship Between Time Between Last Dose and Delivery and Cord:Maternal Anti-Spike (S) Antibody Ratios by Preterm Status, Stratified by Number of Doses

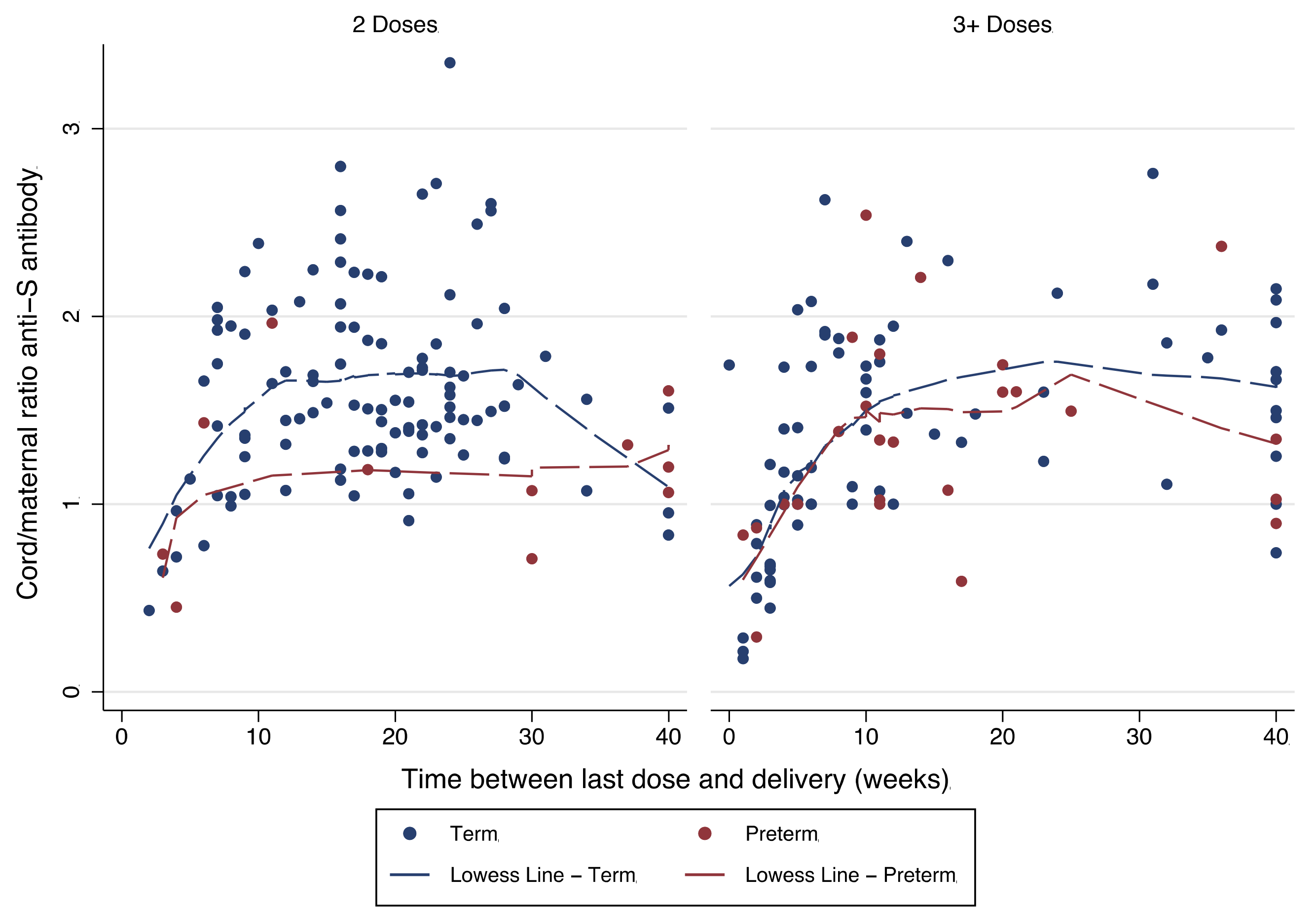


© 2024 Kachikis A et al. *JAMA Network Open.*
