# Supplementary material for: Timing of Maternal COVID-19 Vaccine and Antibody Concentrations in Infants Born Preterm
Source: JAMA Netw Open. 2024 Jan 19;7(1):e2352387. doi: 10.1001/jamanetworkopen.2023.52387 (PMC10799259; doi:10.1001/jamanetworkopen.2023.52387)
Supplement: Supplement 2. — Data Sharing Statement [file jamanetwopen-e2352387-s002.pdf]

Data Sharing Statement

Kachikis. Timing of Maternal COVID-19 Vaccine and Antibody Concentrations in Infants Born Preterm. *JAMA Netw Open*. Published January 19, 2024.
doi:10.1001/jamanetworkopen.2023.52387

Data
Data available: No

Additional Information
Explanation for why data not available: The data associated with this cohort is still under analysis and is therefore not currently available.
